# Elevation of IL-6 and IL-33 Levels in Serum Associated with Lung Fibrosis and Skeletal Muscle Wasting in a Bleomycin-Induced Lung Injury Mouse Model

**DOI:** 10.1155/2019/7947596

**Published:** 2019-03-27

**Authors:** Jiunn-Min Shieh, Hui-Yun Tseng, Fang Jung, Shih-Hsing Yang, Jau-Chen Lin

**Affiliations:** ^1^Division of Chest Medicine, Department of Internal Medicine, Chi Mei Medical Center, Yongkang, Tainan, Taiwan; ^2^Department of Respiratory Therapy, Fu Jen Catholic University, New Taipei City, Taiwan; ^3^Department of Chemistry, Fu Jen Catholic University, New Taipei City, Taiwan; ^4^Graduate Institute of Biomedical and Pharmaceutical Science, Fu Jen Catholic University, New Taipei City, Taiwan

## Abstract

Weight loss due to skeletal muscle atrophy in patients with chronic pulmonary disease is negatively correlated with clinical outcome. Pulmonary fibrosis is a chronic and progressive interstitial lung disease characterized by the dysregulated deposition of the extracellular matrix (ECM) with the destruction of normal tissue, resulting in end-stage organ failure. BLM-induced fibrosis is one of several different experimental models of pulmonary fibrosis, characterized by inflammation and excessive ECM deposition. We directly induced mouse lung injury by the intratracheal administration of bleomycin and monitored the physiological and biochemical changes in lung and skeletal muscle tissues by using lung function testing, ELISA, Western blotting, and immunohistochemistry. Here, we found that BLM-induced lung fibrosis with thickened interstitial lung tissue, including fibronectin and collagen, was correlated with the increased serum concentrations of IL-6 and IL-33 and accompanied by reduced lung function, including FRC (functional residual capacity), C chord (lung compliance), IC (inspiratory capacity), VC (vital capacity), TLC (total lung capacity), and FVC (forced vital capacity) (*p* < 0.05). The activity of AKT in lung tissue was suppressed, but conversely, the activity of STAT3 was enhanced during lung fibrosis in mice. In addition, we found that the amount of sST2, the soluble form of the IL-33 receptor, was dramatically decreased in lung fibrosis tissues. The skeletal muscle tissue isolated from lung injury mice increased the activation of STAT3 and AMPK, accompanied by an increased amount of Atrogin-1 protein in BLM-induced lung fibrosis mice. The mouse myoblast cell-based model showed that IL-6 and IL-33 specifically activated STAT3 and AMPK signaling, respectively, to induce the expression of the muscle-specific proteolysis markers MuRF1 and Atrogin-1. These data suggested that increased levels of IL-6 and IL-33 in the serum of mice with BLM-induced lung injury may cause lung fibrosis with thickened interstitial lung tissue accompanied by reduced lung function and muscle mass through the activation of STAT3 and AMPK signals.

## 1. Introduction

Pulmonary fibrosis is a chronic and progressive interstitial lung disease characterized by the dysregulated deposition of ECM with the destruction of normal tissue, resulting in end-stage organ failure. Idiopathic pulmonary fibrosis (IPF), a progressive disease with poor prognosis, is considered the most common severe form of pulmonary fibrosis, with a median survival of three years after diagnosis and no proven effective therapy [1]. The abnormal fibroblastic proliferation and accumulation of ECM proteins, such as collagen, have been highlighted by recent therapeutic experiments for IPF [2]. Although several potential drugs for IPF treatment, including corticosteroids [3], azathioprine plus prednisolone [4], cyclophosphamide [5], bosentan [6], and interferon (IFN*γ*1b) [7], are in clinical trials, there is no effective pharmacological therapy to improve the survival of patients with IPF [8].

Weight loss due to skeletal muscle atrophy in patients with various diseases, such as cancer cachexia and chronic pulmonary disease, is negatively correlated with clinical outcome [9]. Recent reports have demonstrated that muscle atrophy may be due to muscle protein degradation by the ubiquitin-proteasome system (UPS), which is activated by diverse catabolic stimuli, including growth hormone, cytokines, and nutritional status. Two ubiquitin ligases, muscle-specific RING finger 1 (MuRF1) and Atrogin-1 (FBXO32), are markedly increased in atrophied muscle tissue and serve as muscle-specific proteolysis markers [10]. However, the molecular mechanism modulating muscle loss through the activation of the ubiquitin-proteasome system in patients with chronic lung diseases remains unclear.

IL-6 mediates many inflammatory processes in the lungs, and its dysregulated release has been implicated in the pathogenesis of a variety of respiratory disorders [11, 12]. The cytokine IL-6 is elevated in mice and humans with pulmonary fibrosis [13, 14]. Several genetic studies in both animals and humans have shown the potential association between IL-6 and the development of fibrosis [15, 16]. Blockade of the second increase in IL-6 by IL-6-neutralizing antibody during the fibrotic stage of BLM-induced lung injury can improve lung fibrosis [17, 18]. Many reports have shown that the increased circulation of IL-6 can regulate muscle mass by decreasing protein synthesis and increasing proteolysis during cancer cachexia [19, 20]. Recent studies have shown that STAT3 activation can promote protein degradation and muscle atrophy through the myostatin pathway to induce the expression of Atrogin-1; conversely, inhibition of SAST3 activity may attenuate the loss of muscle mass [21, 22].

IL-33 is expressed in various organ tissues in the human body, including endothelial cells, bronchial cells, and intestinal epithelial cells [23]. Recent studies have demonstrated IL-33 expression in type 2 inflammatory diseases, such as severe asthma [24] and inflammatory bowel disease [25]. IL-33/ST2 signaling is involved in various types of lung disease, including ventilator-induced lung injury (VILI), chronic obstructive pulmonary disease (COPD), and lung fibrosis [26–28]. Studies have demonstrated that IL-33 promotes ST2-dependent organ tissue fibrosis in several animal models, including lung [26], pancreas [29], and liver [30] models.

BLM-induced fibrosis is a common model for the pathogenesis of pulmonary fibrosis, characterized by inflammation and excessive ECM deposition [31]. Recent studies in murine lung injury models support the pathogenic role of an early inflammatory response involving danger signals in the form of uric acid production [32]. In addition, several genes encoding chemokines and cytokines that are not associated with acute inflammatory pathways are upregulated in IPF [33]. Therefore, the selective modulation of key inflammatory pathways has also been proposed as a research focus in future studies [34]. The present study investigated the potential mechanism of cytokines induced by the intratracheal administration of bleomycin and the relationship between lung fibrosis and muscle mass loss based on a lung injury mouse model.

## 2. Methods

### 2.1. Cell Culture

C2C12 mouse adherent myoblasts were obtained from BCRC (Bioresource Collection and Research Center, Hsinchu, Taiwan). The cells were grown in Dulbecco's modified Eagle's medium (DMEM, HyClone, Logan, UT) supplemented with 10% fetal bovine serum (FBS) (HyClone), 1.5 g/L of NaHCO3, 4.5 g/L glucose, 2 mM glutamine, and 1% antibiotics. The cell line was maintained in a 5% CO2 atmosphere at 37°C. The culture medium containing 10% FBS was substituted with 2% horse serum for 72 hours to induce myogenic differentiation of C2C12 cells. The recombinant mIL-6 and mIL-33 were purchased from Cell Guidance Systems (Cambridge, UK).

### 2.2. Animal Model

Ten-week-old male C57BL/6 mice from the National Laboratory Animal Center (Taipei, Taiwan) were used in the present study. The mice were maintained under standard laboratory conditions (temperature: 23-24°C, adequate relative humidity, 15 air changes per hour, and artificial lighting with a 12 h circadian cycle). Pelleted food and tap water were provided ad libitum. All animal procedures were approved by the Institutional Animal Care and Use Committee of Fu Jen Catholic University (IACUC approval number: A10537) and performed in accordance with the Guide for the Care and Use of Laboratory Animals of the institute. All surgeries were performed under anesthesia, and all efforts were made to minimize pain or discomfort in the animals.

### 2.3. BLM-Induced Pulmonary Injury and Fibrosis

At 10 weeks of age, the mice were anesthetized i.p. with a mixture of ketamine (100 mg/kg) (Pfizer, New York, USA) and xylazine (10 mg/kg) (Bayer, Leverkusen, Germany). Lung injury was induced by the instillation of 2 mg/kg body weight of bleomycin (Sigma-Aldrich, St. Louis, MO, USA) in 30 *μ*L of saline into the trachea through the mouth using a laryngoscope and fine tracheal tube [35]. The control group mice were administered 30 *μ*L of normal saline. The anesthetized mice were kept on a warm plate for recovery, and then the mice were maintained under standard laboratory conditions. The experimental animals were monitored daily for adverse clinical signs, including body weight, appearance, hydration status, and any behavioral changes. Mouse body weight was measured every two to three days throughout the entire experiment. In general, the BLM-induced lung injury mouse is predicted to lose between 10% and 20% body weight after BLM exposure but may slightly increase body weight from day 10 [36]. If the experimental mouse lost over 20% body weight and continued to lose weight for two days, the experimental mouse was sacrificed for humane endpoints.

### 2.4. Mouse Sacrifice

The mice were anesthetized (i.p.) with a mixture of ketamine and xylazine. The mice were endotracheally intubated with an airway catheter and connected to a forced pulmonary maneuver system (Buxco research system; Buxco Electronics, Wilmington, NC). The anesthetized animal was imposed with a breathing frequency of 150 breaths/min. The FRC (functional residual capacity) was determined with Boyle's law FRC. The values of lung function, including C chord (lung compliance), IC (inspiratory capacity), VC (vital capacity), TLC (total lung capacity), and FVC (forced vital capacity), were measured using a Buxco research system [37]. After lung function assays, the experimental mice were sacrificed and blood was collected by cardiac puncture.

### 2.5. Bronchoalveolar Lavage (BAL)

After cardiac puncture, the bronchoalveolar lavage fluid (BALF) of experimental mice was obtained via an airway catheter by lavaging with 1 mL of sterile saline [38]. The BALF was centrifuged (250 × *g*, 10 min, 4°C), and the supernatant was aliquoted and stored at -20°C for further protein concentration measurements.

### 2.6. Cytokine Assays

Mouse IL-6 and IL-33 concentrations were determined in serum by using the ELISA Ready-SET-Go Kit (eBioscience) according to the manufacturer's protocols. The absorbance was read at 450 nm using an Epoch ELISA reader (Epoch, BioTek, USA).

### 2.7. Histopathology

The left primary bronchus of lavaged whole lungs was tied with a suture and then excised for Western blot analysis. The right lung lobes were inflated via an airway catheter in 4% buffered paraformaldehyde. The fixed lung specimen was sectioned at a 5 *μ*m thickness for light microscopy. The slides were stained with hematoxylin and eosin or with Masson's trichrome staining. IHC staining was performed to detect fibrinogen and collagen in sections with lung damage.

### 2.8. Preparation of Lung Homogenates

The left lung lobes of mice were sliced and homogenized on ice in lysis buffer (150 mM NaCl, 5 mM EDTA, 50 mM Tris, pH 7.4, 0.02% NaN_3_, 1% Triton X-100, and protease inhibitor cocktail) according to the manufacturer's instructions of Bullet Blender tissue homogenizer (Next Advance Inc., Troy, NY). The homogenates were centrifuged (4°C, 2500 × *g*, 15 min) and stored at -80°C until analysis. The protein concentration of the homogenate was determined by using a bicinchoninic acid (BCA) protein assay kit (Pierce™, Thermo Fisher Scientific).

### 2.9. Western Blotting

Equal amounts of mouse lung homogenate were separated by SDS-polyacrylamide gel electrophoresis (PAGE) for Western blotting with specific antibodies against fibronectin (Millipore, Billerica, MA), Atrogin-1, MuRF1 (Abcam, Cambridge, MA, USA), GAPDH, ST2 (GeneTex Inc, Irvine, CA), *α*-tubulin (Santa Cruz), p-STAT3 (Tyr705), STAT3, p-AMPK*α* (T172), AMPK*α*, p-AKT (S742), and AKT (Cell Signaling, Beverly, MA, USA).

### 2.10. Statistical Methods

The quantitative data were expressed as the mean ± SD. Statistical significance of differences between groups was analyzed using the *t*-test. *p* < 0.05 was considered to be significant.

## 3. Results

### 3.1. Lung Function Was Reduced in Mice with BLM-Induced Fibrotic Lung

**Figure 1:**
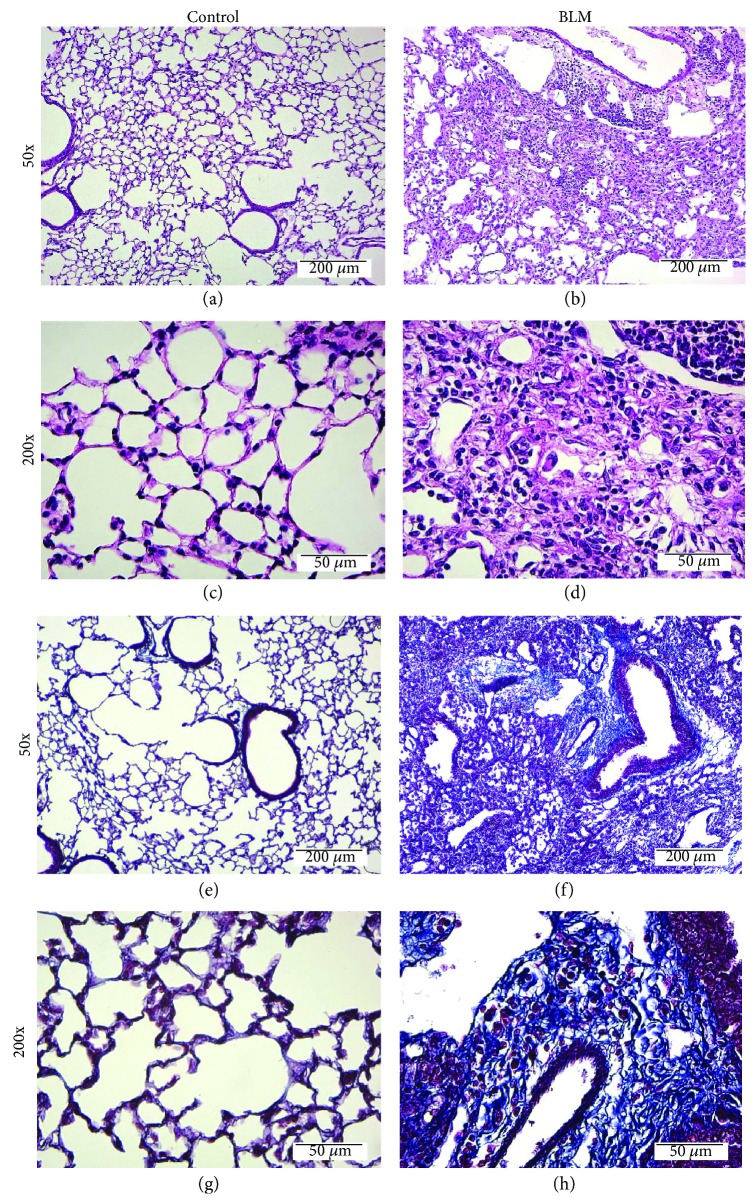
BLM-induced lung fibrosis in mice. C57BL/6 mice were intratracheally administered bleomycin (2 mg/kg) for 14 days. (a–d) Lung H&E staining. (e–h) Masson's trichrome staining of lung tissue from (a, c) control mice and (b, d) BLM-induced lung fibrosis mice. The photos in (a, b, e, and f) represent 50x magnification. The photos in (c, d, g, and h) represent 200x magnification. The collagen in lung fibrosis tissue was stained blue by Masson's trichrome staining. Bar = 200 *μ*m at 50x magnification and 50 *μ*m at 200x magnification as indicated.

**Figure 2:**
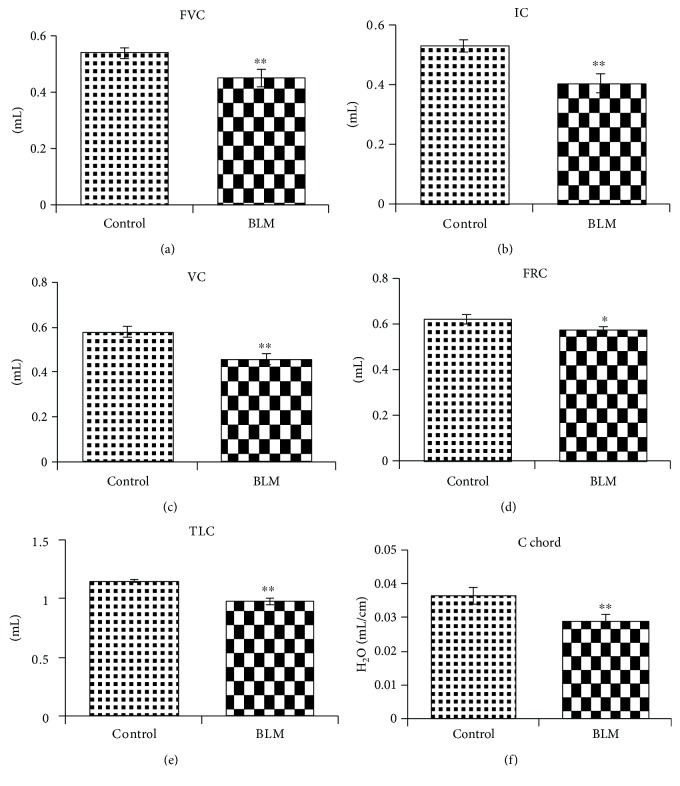
Lung function was reduced in mice with BLM-induced fibrotic lungs. Anesthetized mice were endotracheally intubated with an airway catheter, and the parameters of lung function were measured by a pulmonary function testing system. (a) FVC (forced vital capacity), (b) IC (inspiratory capacity), (c) VC (vital capacity), (d) FRC (functional residual capacity), (e) TLC (total lung capacity), and (f) C chord (lung compliance). The quantitative data were expressed as the means ± SD. Light spots represent the lung function of control mice (*n* = 4), and dark spots represent the lung function of mice with BLM-induced lung injury (*n* = 5). ^∗^*p* < 0.05 and ^∗∗^*p* < 0.005 compared with control mice.

Lung injury in C57BL/6 mice was induced through the intratracheal administration of bleomycin (2 mg/kg) on day 0, and control mice were treated with normal saline. To evaluate the status of BLM-induced lung injury in these mice, histological examination of lung specimens was performed by H&E or Masson's trichrome staining. The results demonstrated that the intratracheal administration of bleomycin induced thickening of the major tracheal wall with accumulation of fibroblasts and immune cells in interstitial lung tissues by day 14 (Figures 1(a)–1(d)). Masson's trichrome staining of lung tissue showed that bleomycin could induce lung fibrosis in mice with a higher collagen content (blue region) in mesenchymal tissue compared with that of the lung tissue in control mice (Figures 1(e)–1(h)). To confirm whether the injury led to lung fibrosis, the lung function of mice was measured by the Buxco® pulmonary function testing system. The results showed that the lung function of mice with BLM-induced lung fibrosis was significantly decreased, including FRC (functional residual capacity), C chord (lung compliance), IC (inspiratory capacity), VC (vital capacity), TLC (total lung capacity), and FVC (forced vital capacity), when compared with that of the control group. This finding indicated that lung function was reduced after BLM-induced lung injury (Figure 2).

### 3.2. BLM-Induced Lung Injury May Cause Body Weight Loss and Muscle Atrophy

**Figure 3:**
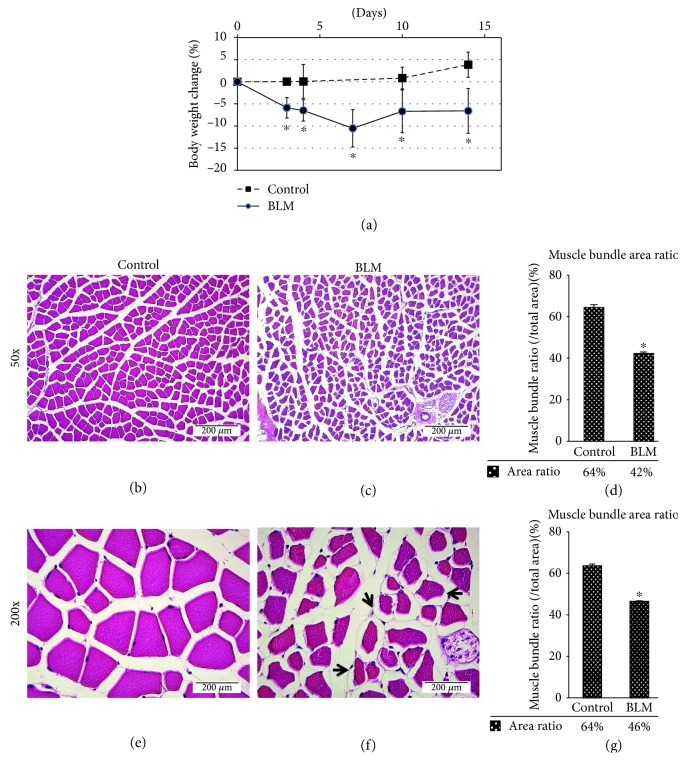
BLM-induced lung injury may cause body weight loss and muscle atrophy. (a) The body weights of experimental mice were measured every two to three days during the experimental periods. Square boxes represent the body weight change of control mice (*n* = 6), and dots represent the body weight change of mice with BLM-induced lung injury (*n* = 5). The quantitative data were expressed as the means ± SD. ^∗^*p* < 0.05 compared with control. (b–g) Cross-sectional H&E staining of quadriceps muscle in (b, e) control mice and (c, f) mice with BLM-induced lung fibrosis. The photos represent 50x magnification in (b–d) and 200x magnification in (e–g). The quantitative analysis of the muscle bundle area to the total area in photos is shown in (d, g) (*n* = 3). The values of all data are averages from at least three photos with different fields of view. The muscle bundle area was measured by ImageJ software. ^∗^*p* < 0.05 compared with control mice. Bar = 200 *μ*m at 50x magnification and 50 *μ*m at 200x magnification as indicated. The black arrow indicates the detached basal lamina.

The body weight change of mice with BLM-induced lung injury and fibrosis was measured every 2-3 days during the experimental period. The results showed that the body weight of BLM-induced mice gradually decreased by as much as approximately 10% of the body weight by day 14 compared to that of the control mice (Figure 3(a)). Histological examination of the quadriceps muscle in BLM-induced lung fibrosis mice revealed pathological changes, including variation in fiber size with many small atrophic fibers and separation of the basal lamina from the sarcolemma, when compared to the control mice (Figure 3(b)). To quantify the intensity of muscle atrophy in the quadriceps muscle of mice, the cross-sectional area of the quadriceps muscle bundle (medial region) on the H&E staining image was calculated by ImageJ software. The results showed that the ratio of the muscle bundle area to the total muscle tissue area was dramatically decreased from 64% in control mice to 42% in lung injury mice. This finding indicated that BLM-induced lung injury may lead to quadriceps muscle atrophy and muscle mass loss through the circulation of cytokines secreted from injury tissues.

### 3.3. The Circulation of IL-6 and IL-33 Was Increased in Mice with BLM-Induced Lung Fibrosis

**Figure 4:**
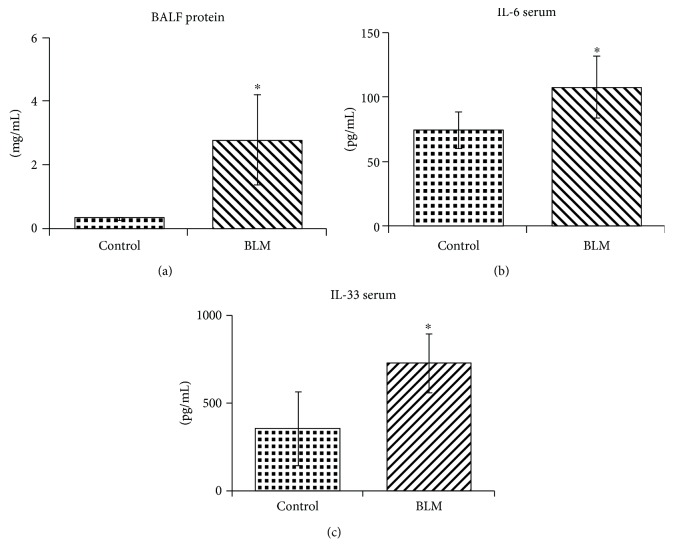
The circulation of IL-6 and IL-33 was increased in mice with BLM-induced lung fibrosis. (a) Protein levels in bronchoalveolar lavage fluid (BALF) were estimated by using a BCA kit assay. The cytokines in mouse serum were measured by ELISA. (b) IL-6. (c) IL-33. Light spots represent the cytokine amount in control mice (*n* = 4), and dark spots represent the cytokine amount in mice with BLM-induced lung injury (*n* = 5). The quantitative data were expressed as the means ± SD. ^∗^*p* < 0.05 compared with control.

The BALF protein level in the BLM-induced lung injury was significantly increased when compared to the level in the control mice (Figure 4(a)), indicating that the increased vascular endothelial permeability may be due to the secretion of inflammatory cytokines from the injured lung tissue. To ascertain the relationship between lung injury and muscle wasting, the cytokines in the serum of mice were measured by ELISA. The lung fibrosis-related cytokines IL-6 and IL-33 were significantly increased in the blood of mice with BLM-induced lung injury by day 14 compared with the control mice, indicating that IL-6 and IL-33 may associate with lung injury and muscle wasting in BLM-induced lung fibrosis mouse models (Figures 4(b) and 4(c)).

### 3.4. Activation of STAT3 Is Associated with Inactivation of AKT in Fibrotic Lung Tissue

**Figure 5:**
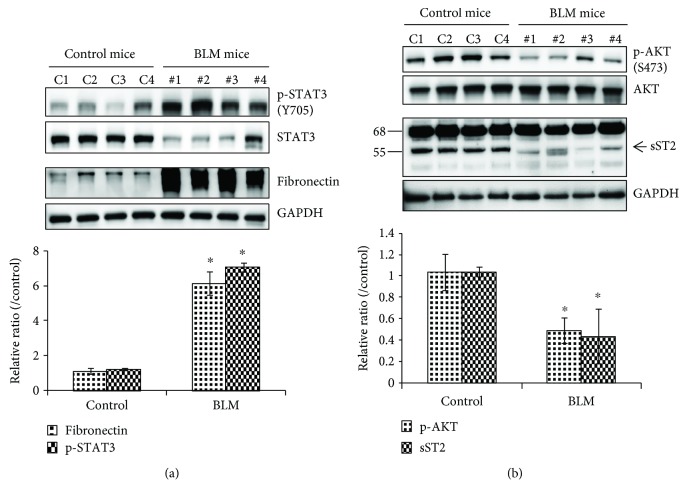
Activation of STAT3 is associated with inactivation of AKT in fibrotic lung tissue. The sliced left lung lobes of mice were homogenized, and the lysate was analyzed by Western blotting. (a) Fibronectin and STAT3. (b) ST2and AKT were detected in lung lysates. The intensity of bands in the Western blots was measured by ImageJ software. GAPDH was used as an internal control. The quantitative data were expressed as the means ± SD. ^∗^*p* < 0.05 compared with control.

Western blot analysis of lung tissues in BLM-induced lung fibrosis mice showed an increased expression of extracellular matrix proteins, such as fibronectin, and phosphorylation of STAT3 (Tyr705) protein, a downstream mediator of IL-6 signaling, when compared with lung tissues of the control mice (Figure 5(a)). In addition, decreased expression of the soluble form of ST2 (55 kDa), the receptor of IL-33, and decreased phosphorylation of AKT (S742) protein were found in the lung tissue of mice with BLM-induced lung fibrosis, indicating that lung tissue cells were affected by circulating IL-6 and IL-33 during lung fibrosis (Figure 5(b)).

### 3.5. IL-6 and IL-33 May Synergistically Cause Muscle Atrophy

**Figure 6:**
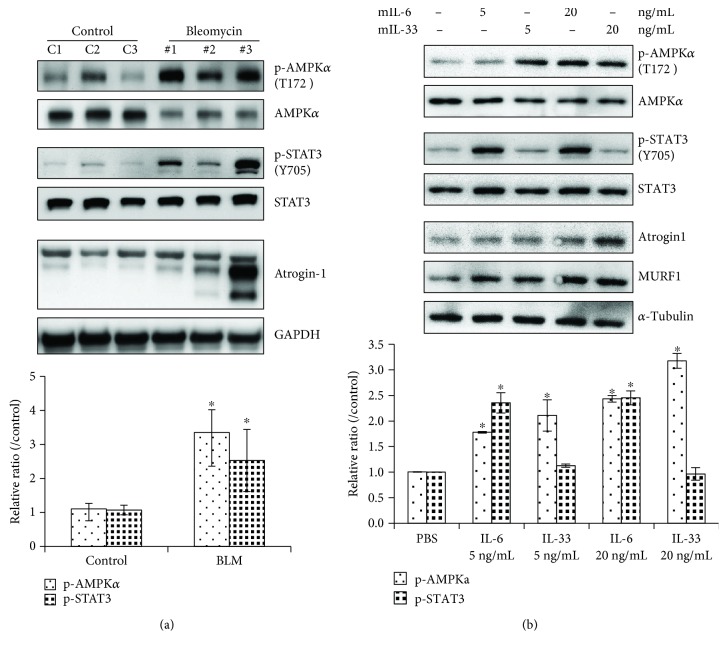
IL-6 and IL-33 may synergistically cause muscle atrophy. (a) The quadriceps muscle of the mice was homogenized, and the lysate was analyzed by Western blotting with specific antibodies against STAT3, AMPK, and Atrogin-1. (b) C2C12 cells, mouse adherent myoblasts, were incubated with 2% horse serum for 72 hours and stimulated with recombinant mouse IL-6 and IL-33 in serum-free medium as indicated for 24 hours. The remaining cells were harvested, and the levels of p-STAT3, STAT3, p-AMPK*α*, and AMPK*α* in the cell lysate were analyzed by Western blotting. *α*-Tubulin and GAPDH were used as internal controls. The intensity of bands in the Western blots was measured by ImageJ software. The quantitative data were expressed as the means ± SD. ^∗^*p* < 0.05 compared with control.

**Figure 7:**
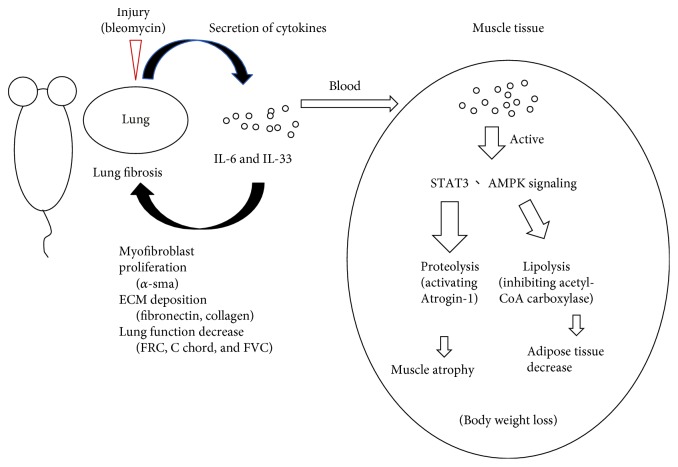
Increased levels of IL-6 and IL-33 in the serum of mice with BLM-induced lung injury may cause body weight loss through the activation of STAT3 and AMPK signals. Schematic representation of the role of IL-6 and IL-33 in the BLM-induced lung fibrosis mouse model. IL-6 and IL-33 are secreted from inflammatory lung tissue and modulate lung remodeling, including myofibroblast proliferation, ECM deposition, and decreased lung function. In addition, IL-6 and IL-33 synergistically cause body weight loss in mice through the activation of STAT3 and AMPK signaling to induce the expression of proteolysis- and lipolysis-related genes.

To determine whether skeletal muscle atrophy was modulated by IL-6 or IL-33 in serum secreted from the injured lung tissue, the skeletal muscle tissue was excised and analyzed by Western blotting. The Western blot results showed that Atrogin-1, a muscle-specific proteolysis marker, was generally increased in the muscle tissue of mice with BLM-induced lung fibrosis, which is associated with an increase in the activity of STAT3, a downstream mediator of IL-6 signaling, and AMP-activated protein kinase (AMPK), an important regulator of energy metabolism (Figure 6(a)). To confirm whether the activation of STAT3 and AMPK was mediated by the circulation of the inflammatory cytokines IL-6 and IL-33, C2C12 mouse adherent myoblast cells were treated with recombinant mIL-6 and mIL-33 to detect the effect of these cytokines on myocyte-like cells. The results showed that mIL-6 can activate STAT3 signaling and that mIL-33 can activate the phosphorylation of AMPK (T172). In addition, both cytokines can also induce the expression of Atrogin-1 and MuRF1, suggesting that BLM-induced lung fibrosis in mice may influence the proteolysis of long-distance muscle tissues through the circulation of both IL-6 and IL-33 secreted from injured lungs during lung fibrosis (Figure 6(b)). In conclusion, lung injury induced by bleomycin caused lung damage and inflammation with the secretion of IL-6 and IL-33 in blood, which modulated lung remodeling, including myofibroblast proliferation, ECM deposition, and decreased lung function. In addition, IL-6 and IL-33 may affect the metabolism status of whole body tissues, such as adipose and muscle tissues, through the activation of STAT3 and AMPK signaling, which leads to lipolysis and proteolysis, and both ultimately cause body weight loss and skeletal muscle mass loss by inhibiting acetyl-CoA carboxylase (ACC) and activating Atrogin-1 protein, respectively (Figure 7).

## 4. Discussion

The present study provides important evidence of body weight loss and skeletal muscle atrophy in BLM-induced lung fibrosis by the secretion of cytokines from damaged lung tissue. We directly induced mouse lung injury by the intratracheal administration of bleomycin and monitored the levels of the cytokines IL-6 and IL-33 secreted from damaged lung tissue into the blood. The circulation of cytokines may affect the metabolism of skeletal muscle and induce muscle atrophy via STAT3 and AMPK signaling.

The cause of muscle dysfunction with lung fibrosis is multifactorial, including hypercapnia, malnutrition, systemic inflammation, and oxidative stress. BLM-induced lung injury and subsequent lung fibrosis with restrictive lung disease can reduce lung function, including FRC (functional residual capacity), C chord (lung compliance), IC (inspiratory capacity), VC (vital capacity), TLC (total lung capacity), and FVC (forced vital capacity). This effect may decrease the exchange rate of oxygen and induce the accumulation of carbon dioxide in blood. A recent report showed that high carbon dioxide levels in the blood might activate AMPK and then induce skeletal muscle atrophy [39]. These results indicated that BLM-induced lung fibrosis in mice may modulate metabolism and muscle atrophy by regulating AMPK activity. Although we also found that the increasing activity of AMPK in skeletal muscle may be due to the effect of IL-33 in serum, we could not rule out the activation of AMPK caused by hypoxia because of reduced lung function in lung fibrosis mice.

Gilhodes et al. showed that a dose-dependent reduction of body weight was observed in BLM-induced lung fibrosis mice treated with higher BLM concentrations, including 0.5, 0.75, and 1.0 mg/kg, accompanied by a significant decrease in dynamic lung compliance and forced vital capacity [40]. In addition, the weight loss of mice after BLM exposure (3.0 mg/kg) decreased by nearly 10% from day 2 and then gradually thereafter. Conversely, the BLM-treated mice slowed weight loss and underwent weight gain from approximately day 10 [36]. We thought that this phenomenon may be due to lung tissue recovery. In this study, a similar phenomenon was observed in which BLM-treated mice increased in body weight from day 10 and subsequently gradually increased thereafter. The present data showed that the highest level of IL-6 in the serum of a BLM-treated mouse with the highest weight loss was associated with the highest activation of STAT3 signaling and high expression of Atrogin-1 protein in the quadriceps muscle tissue. The myocyte-like cell-based model assay also showed that IL-6 stimulation in cells can activate STAT3 signaling. These findings implied that IL-6 may play an important role in the pathogenesis of BLM-induced lung injury leading to muscle wasting. Our data also supported that IL-6 may be an important marker and target cytokine for preventing body weight loss and skeletal muscle atrophy in the clinical care of several chronic lung diseases, including COPD and pulmonary fibrosis. However, the effect of IL-6 cytokine on the expression of Atrogin-1 in skeletal muscle tissue remains unclear.

The current results also showed that IL-33 expression was increased in serum during BLM-induced lung fibrosis, consistent with the findings of previous studies. However, we also found that soluble ST2 was expressed in normal lung tissue of mice but was dramatically reduced in BLM-induced lung injury tissue. This finding is similar to that of a previous study showing the decreased expression of soluble ST2 in ventilator-induced lung injury, which may potentially serve as a new biomarker [28]. Recent studies have shown that IL-33 can communicate between the nervous and immune systems within the muscle tissue to medicate muscle regeneration by regulating muscle Treg cell homeostasis in young mice [41]. These results implied that IL-33 may be associated with the loss of skeletal muscle in aging mice. Our studies also showed that the secretion of IL-33 in injured lung tissue may be associated with the loss of skeletal muscle in inflammatory lung tissue through activation of the AMPK signaling pathway. However, the detailed molecular mechanism of IL-33 mediating weight loss and skeletal muscle atrophy through regulating whole-body metabolism during lung injury, including VILI, chronic COPD, and lung fibrosis, remains unknown. In the current study, we could not clarify whether the origin of the IL-33 cytokine is produced by lung inflammatory infiltration or how many other cytokines could additionally activate AMPK signaling. Therefore, these questions will be addressed in future studies.

AMP-activated protein kinase (AMPK) is an important regulator of whole-body energy metabolism that mediates energy homeostasis, including carbohydrate, lipid, and protein metabolism. The dysregulation of AMPK causes obesity, metabolic syndrome, cardiovascular disease, and cancer [42]. Greer et al. showed that AMPK can directly regulate the FOXO3 transcription factor, which induces atrophy-related ubiquitin ligase Atrogin-1 and leads to skeletal muscle atrophy [43]. Another study showed that inhibition of PI3K/AKT activation can lead to activation of FOXO transcription factors and Atrogin-1 induction; conversely, AKT overexpression inhibits FOXO and Atrogin-1 expression [44]. In this study, we found that AKT3 activity was suppressed but AMPK was activated in both lung tissue and skeletal muscle tissue of mice with BLM-induced lung fibrosis, which indicates that lung tissue and muscle tissue cells were affected by circulating cytokines during lung fibrosis to mediate muscle mass through the activation of the FOXO transcription factor. In addition, the present results also showed that mIL-33 could activate AMPK signaling, which promotes the catabolic process, including proteolysis and lipolysis in cell models, indicating that the secretion of IL-33 due to lung injury may activate catabolic signaling in muscle tissue to provide energy through inflammatory cytokines. However, there are no studies regarding the relationship between IL-33 and AMPK in patients with chronic lung fibrosis. Although the loss of body weight and muscle atrophy in chronic lung disease is a multifactorial event, these accumulating data suggest that both mIL-6 and mIL-33 are increased in serum during lung fibrosis, which may synergistically cause body weight loss in mice through the activation of STAT3 and AMPK signaling to induce the expression of proteolysis- and lipolysis-related genes. Therefore, we think that attenuating the secretion of IL-6 and IL-33 from damaged lung tissue during lung injury might prevent body weight loss and skeletal muscle atrophy, thus improving the clinical outcome.

## 5. Conclusions

These data suggested that increased levels of IL-6 and IL-33 in the serum of mice with BLM-induced lung injury may cause lung fibrosis with thickened interstitial lung tissue accompanied by reduced lung function and muscle mass through the activation of STAT3 and AMPK signals.

## Data Availability

All data generated and analyzed during the study are included in the published article and can be shared upon request.

## Abbreviations

BLM: Bleomycin
ECM: Extracellular matrix
FRC: Functional residual capacity
C chord: Lung compliance
IC: Inspiratory capacity
VC: Vital capacity
TLC: Total lung capacity
FVC: Forced vital capacity
IPF: Idiopathic pulmonary fibrosis
MuRF1: Muscle-specific RING finger 1
BALF: Bronchoalveolar lavage fluid
STAT3: Signal transducer and activator of transcription 3
AMPK: 5′AMP-activated protein kinase
UPS: Ubiquitin-proteasome system
BCA: Bicinchoninic acid.
